# Incentives and disincentives to private sector reporting on family planning in Kenya: *why these matter, and how they may change over time*

**DOI:** 10.12688/gatesopenres.13909.2

**Published:** 2023-08-07

**Authors:** Gabrielle Appleford, Daniel Mumbia, Priya Emmart

**Affiliations:** 1 Track20, AVENIR, Nairobi, Kenya, Kenya; 2 Track20, AVENIR, Glastonbury, Connecticut, USA

**Keywords:** Health information policy, private sector, family planning

## Abstract

**Background:** This study sought to understand private sector reporting on family planning in Kenya’s health information system (KHIS). We approached this through three lenses: governance, procedural and technical. Our study looked at these areas of interest in Kenya, complemented by deeper exploration in Nairobi County.

**Methods: **The study used mixed methods drawing on analysis from the KHIS and surveys, complemented by desk review. The qualitative research entailed group discussions with public sector personnel while more in-depth qualitative interviews were done with public and private sector respondents. A framework matrix was developed for the qualitative analysis. The study was approved by the Ministry of Health in March 2022 and conducted over the period March - May 2022.

**Results: **From a governance lens, private sector respondents recognised the importance of registry and reporting as a government policy requirement. From a procedural lens, private sector respondents saw reporting procedures as duplicative and parallel processes as reports are not generated through digitised information systems. From a technical lens, private sector reporting rates have improved over time however other data quality issues remain, which include over- and under-reporting of family planning services into KHIS. Secondary analysis for Nairobi County shows that the private facility contribution to family planning has declined over time while family planning access through pharmacies have grown over the same period; there is no visibility on this shift within the KHIS. Changes in private sector family service provision have implications for assumptions underpinning modern contraceptive modelled estimates and programmatic decision-making.

**Conclusions:** There is limited monitoring of the incentives and disincentives for reporting by private health facilities into the KHIS. These have changed over time and place. Sustained private sector engagement is important to align incentives for reporting as is greater visibility on the role of pharmacies in family planning.

## Introduction

Like many other lower- and middle-income countries (LMICs), Kenya has a pluralist health system in which a mix of public and private entities deliver health related goods and services. Within the health sector, interactions between public and private entities are intended to improve health system performance in line with policies established by government. Data and information are foundational to interaction and performance. However too often data and information are incomplete making them unreliable for performance monitoring.

This study sought to understand private sector reporting on family planning in Kenya’s health information system (KHIS). We approached this through three lenses: governance, procedural and technical. Specifically, we were interested in exploring:

- Governance: What are the reporting incentives and disincentives for private sector entities delivering family planning services? Have these changed over time?
- Procedural: How do private sector entities report into the KHIS? Are there concerns with the quality of reporting? Has this changed over time?
- Technical: What is the contribution of the private sector to family planning? What are the assumptions underpinning adjustments in modelled estimates of contraceptive prevalence? Do these remain valid?

Our study looked broadly at these areas of interest in Kenya, complemented by a deeper exploration in Nairobi County.

## Methods

### Design

This study used a mixed methods approach drawing on analysis from the KHIS and population-based surveys, complemented by qualitative research. The qualitative research entailed group discussions with county health teams in seven counties. More in-depth qualitative interviews were done in Nairobi County with public and private sector respondents. A desk review was conducted as part of this study and included background articles relevant to the context.

For the purposes of this study, the private health sector is understood as all entities involved in the provision of health care that are neither owned nor directly controlled by government. In the context of Kenya, they include for-profit and not-for-profit entities both local and international, including primary care clinics, maternity/nursing homes, pharmacies (in their service extension role) and hospitals. It does not include other private entities operating along the healthcare value chain. The public health sector is understood as including the Ministry of Health, the county departments of health (CDoH) and service delivery provision through facilities directly owned and operated by government.

### Health information systems and population-based surveys

Data on reporting rates by sector were extracted from the KHIS. The KHIS is the system used in Kenya to integrate data collection, processing, and reporting of routine service statistics and other health information for the purposes of monitoring service delivery performance. Data on facilities by ownership were extracted from the Kenya Health Master Facilities List (KHMFL). The KHMFL is an application which contains all registered health facilities that are identified with a unique code and provides details on geographic location, administrative location, ownership, type and the services offered.

Secondary data analysis was conducted for the 2014 Demographic and Health Survey (DHS) and 2016, 2017, 2018, 2019, and 2020 Performance Monitoring for Action (PMA) surveys. For women who were using a modern method and residing in Nairobi, weighted distributions were created of the source of modern methods of family planning (in total and by method) for each survey. Results are limited to methods with 25 (unweighted) observations.

### Group discussion and individual interviews

Group discussions with reproductive health coordinators and health records and information officers (HRIOs) were conducted in seven counties (Laikipia, Nyandarua, Isiolo, Mombasa, Meru, West Pokot, Nakuru). Counties, 47 in total, are the second level of government in Kenya. Counties were purposively selected as part of a Track20/MoH family planning training activity. This was a scheduled 30-minute discussion in the training and entailed 15–20 participants per group per county (approximately 120 participants). Discussions were used to verify county health team perceptions of the growth of the private sector and conformity with reporting requirements in their context.

In-depth interviews were conducted with reproductive health coordinators and/or HRIOs from four of Nairobi’s 17 sub-counties, managers from two international family planning organisations, and three private providers, affiliated with one of the family planning organisations, through social franchising. The four Nairobi sub-counties were selected based on contextual factors, such as the presence of large informal settlements and family planning organisations. Selection of sub-county and non-governmental respondents was done in conjunction with the Nairobi Reproductive Health Coordinator. Private sector respondents were identified by the family planning organisation. In total, 11 respondents were interviewed.

Group discussions were led by the second author with the primary author participating in three county group discussions to test the guide and as part of quality assurance. The in-depth interviews were conducted by both the primary and secondary authors. Respondents were given the option of an in-person or remote interview using an online meeting application. All those that provided consent to be interviewed, opted for an online interview citing convenience as the reason. Verbal consent was obtained for the discussions and interviews. Interviews were audio recorded.

### Ethics and consent

The study was approved by the Ministry of Health (MoH), Office of the Director General in March 2022 (reference MoH/ADM/1/1/82 (182)) and conducted over the period March - May 2022. Informed verbal consent was sought for the interviews as the nature of the inquiry presented minimal risk to participants. Respondents’ information was anonymised as part of data analysis and presentation of findings. Quotations are referenced as county respondent, non-government respondent and private sector respondent respectively.

### Analysis

Detailed notes were prepared for both the group discussions and in-depth interviews by the primary and secondary authors. A coding frame was developed for data extraction, based on the semi-structured group discussion and interview guides. Key themes analysed were the public-private mix of health entities; volumes of family planning services in the private sector; private sector reporting trends; data quality and procedural issues with reporting; use of family planning data, as well as insights or recommendations on how to improve private sector reporting.

Information from the discussions and interviews was triangulated with MoH information systems and was done by the second author. For example, county respondent views on growth of the private sector were triangulated through the KHIS and KHMFL, while volumes of family planning services offered through the private sector were verified through the KHIS. The KHIS provided information on trends over time (a five-year horizon is available) however the KHMFL does not allow for year-on-year comparison in health facility composition and growth. Secondary data analysis using population-based surveys explored the extent and changes in the use of private sector entities by family planning method over time in Nairobi County. This was done by a Track20 expert (see acknowledgements) to provide information on the contribution of private pharmacies to family planning volumes, given that this is not captured in MoH information systems.

A framework matrix was developed by the primary author for the analysis using Microsoft Excel 2016. The matrix was constructed horizontally with the key themes and vertically by respondent. Group discussion and interview notes were condensed, with information arising from data sources inserted into the matrix. Quotes from the detailed notes were inserted as part of data extraction. Where needed, the primary and secondary authors compared notes and understandings to ensure completeness of information and consistency of interpretation. The completed framework matrix was reviewed by all authors. The primary author drafted the paper, and the other two authors reviewed the drafts and final manuscripts.

### Research team and reflexivity

The primary author is a public health specialist with extensive contextual experience, subject matter expertise and qualitative research skills. She is a contracted Track20 consultant based in Nairobi, Kenya. The second author is a Monitoring and Evaluation Officer (MEO) with the Track20 project seconded to the MoH health informatics division, and is based in Nairobi, Kenya. He has contextual experience, and an advanced degree in health informatics. The third author is the Deputy Director of the Track20 project and has extensive expertise in monitoring and evaluation and mixed methods. 

## Results

### Governance

***What are the reporting incentives and disincentives for private sector entities delivering family planning services? Have these changed over time?***

 

*Summary*

Private sector respondents recognised the importance of registry and reporting as a Government of Kenya policy requirement

Public sector respondents indicated that most health facilities in their counties comply with registry and reporting requirements

Public and private sector respondents recognised the limited visibility of pharmacies to family planning services as these entities do not report into the KHIS

There has been substantial growth in both the public and private sectors over time ( Table 1) ^1^ however, this has not kept pace with population growth. Coverage of health facilities remains inadequate and uneven – at its extremes, Nairobi County contains just over 10% of all health facilities, while Isiolo County in the arid lands of northern Kenya has 0.5% ^1^. The health sector as a whole is characterized as one of increasing demand for health care alongside under resourcing, underdeveloped infrastructure, shortages in human resources for health, and essential drugs and medical supplies with significant variation across the country ^2^.

**Table 1.** Facility distribution by ownership (Source: KMHFL).

Year	Private (for-profit)	Private (faith-based)	Total Private	Total Public
2006	2217	792	3009	2120
2013	3500	1053	4553	3965
2022	6004	1140	7144	7204
% growth over time	63%	30%	58%	71%

Devolution, which commenced in 2013, transferred responsibility for service delivery to Kenya’s 47 counties. As part of devolution, the majority of Kenya’s public health facilities and stewardship (oversight) of private facilities was transferred to county departments of health (CDoH). The national MoH retains the roles of health policy and standards formulation, pre-service training for health workers, and management of national referral services as part of health governance ^3^. This includes information and data architecture through the KHIS as well as a digital registry of all health facilities in the KMHFL.

Kenya’s health policy 2014– 2030 aims to strengthen the KHIS for complete and timely reporting of all health-related events, including family planning. The Health Act 2017 and KHIS policy directs all private sector entities to mandatorily submit their data to the MoH, through the CDoH. The Health Act also requires that all private facilities are licensed and registered. This includes inspection, geo-referencing, and registry in the KMHFL. Across counties, respondents indicated that the majority of private health facilities are registered and included within the KMHFL.

*“We have more facilities that what is in the KMHFL, some which are dormant and not reporting but 75% are reporting. New facilities always come to us for mapping i.e., KMHFL…some, months are busy, we get five facilities while others are quiet.”* (County respondent)

Most county respondents indicated that the number of private sector entities had grown over time. Growth has been uneven across counties and more pronounced in urban counties, such as Nairobi and Mombasa where the private sector accounts for 75% and 74% of all health facilities. There was also reportedly substantial growth in private pharmacies, however data and information are lacking as they do not report into the KHIS while only some are registered in the KMHFL.

*“We had a partner who came through national level. We tried to incorporate some chemists [pharmacies] in our system. It was not possible for them to report. Our mothers are still going there for family planning services.”* (County respondent)

There was reported to be a visible shift to more “modernized” private health entities, including franchise chains, alongside more traditional small-to-medium (SME) clinics and nursing homes. Modernisation of the sector was attributed to population demand and improved access, through facility accreditation in universal health care (UHC) schemes, such as the National Health Insurance Fund (NHIF). As part of modernisation in the sector, there was reported to be an increase in the use of digitized information systems and reporting within private sector entities, particularly in urban counties.

*“There is a trend towards modernisation within private facilities and digitised reporting is a requirement of the NHIF and private insurers.”* (Non-governmental respondent)

### Procedural

***How do private sector entities report into the KHIS? Are there concerns with the quality of reporting? Has this changed over time?***

 

*Summary*

Monthly reporting into the KHIS is manual using paper-based tools, submitted in-person to the sub-county by private and public sector entities

Private sector respondents saw current procedures as duplicative and parallel processes as reports are not generated through digitised information systems

Submission of reports provides opportunity to access routine vaccines and family planning commodities however family planning supply is often limited to short-term methods and disrupted due to stock outs

Paper-based reporting formats are seen as necessary for data quality audits and a check against data manipulation

Public and private health facilities provide family planning as part of an essential package of reproductive and child health services. Private entities, along with their public facility counterparts, are expected to produce and submit in-person monthly reports as well as maintain the MoH 512 register, where all client visit details are recorded for reproductive and child health services. The MoH 512 register is the data source for the reports: family planning visits are tallied in the MoH 711 report while commodities distributed to clients are tallied into the MoH 747A Facility Contraceptive Consumption Data Report and Request (FCDRR).

- **MoH 711 Integrated Summary Report Form: reproductive and child health, medical and rehabilitative services**. There are 11 service areas and 157 data elements contained within the form. For family planning, data elements include services offered to clients, including type of contraceptive received, whether it was a new visit or a follow-up visit, whether the service was post-partum or post-abortion as well as information on client age.

- **MoH 747A FCDRR**. This tool collects information on commodities by method—combined oral contraceptive pills, progestin-only pills, injectables, implants (1-Rod), implants (2-Rod), emergency contraceptive pills, IUCDs, male condoms, female condoms, cycle beads, and others.

Digitised information systems within private entities have not facilitated reporting into the KHIS, the generation of MoH monthly reports or maintenance of paper-based registers. Private sector respondents saw manual reporting as duplicative and parallel processes to their digitised systems. For their part, public sector respondents viewed paper-based reporting formats as necessary for data quality audits and a check against data manipulation. There are no immediate plans to replace paper-based reports with electronic reports although there is longer-term work on a national client medical records (CMR) system. There is also a small pilot with a subset of health facilities with CMR directly linked to the KHIS.

*“Reporting is done manually in the 711 and FCDRR; this is then hand-carried to the sub-county HRIO. Providers complain about the time required for reporting, including the need to wait at the sub-county to submit the reports to the HRIOs and collect commodities…this can be a 1–3 hour wait.”* (Non-governmental respondent)

*“The manual system…the entire process is slow. I have to do double, fill in the manual forms from our computerized system.”* (Private sector respondent)

Availability of the registers and reporting forms is a challenge in counties, which often lack adequate tools for facilities (both public and private). Updates to the register and reports to cater for additional reporting elements was undertaken in late 2020 but many facilities are not using these. *“Now we do not have 711 tools even a copy…since August [2021] we have not had 711.”* (County respondent). In some instances, private facilities are advised to make photocopies of the forms using their own resources which further acts as a disincentive for reporting.

*“It’s a cumbersome way of doing it [reporting]…paperwork can have many errors. It’s not easy to correct errors you have made. If the HRIO notices any error during reporting, you have to go back to the facility, write again and take it on different day.”* (Private sector respondent)

Sub-county HRIO have the mandate to give access rights to private sector entities for reporting directly into the KHIS. For a facility to be given access rights, staff capacity must be vetted by the sub-county HRIO. In practice, access rights appear to be granted in very few cases, including for monitoring officers in international non-governmental family planning organisations. This also means that entities reporting into KHIS cannot verify that their facility data has been correctly captured in the system. In some instances, it may not be captured at all. One facility indicated that three years of 711 reports (2016–2019) had not been captured in KHIS. This came to light during a training facilitated by the national KHIS team, after which access rights were subsequently granted to the private entity. This appears to be a rare occurrence, however, and may be due to a lack of procedural clarity. There is also a disincentive to pursue access rights as this does not negate the need for physical submission of hard-copy reports.

*“We do not have access to KHIS system. I can only do manual analysis since I do not have access to the system. I am not able to confirm what I report is captured in DHIS2 [KHIS]. I was given information on how to get access [KHIS] but I did not proceed to follow up since they only insist on sending hard copies.”* (Private sector respondent)

When reports are submitted, private health entities, through their facility-in-charge or designate, can access family planning commodities and vaccines for routine immunisation. These products are distributed through the Kenya Medical Supplies Authority (KEMSA), the government body responsible for procurement, warehousing and distribution of medical supplies and commodities to county and sub-county stores. This provision requires that the private health entity is included in the KMHFL, complies with reporting requirements, and there is ‘excess’ commodity availability beyond what is required in the public sector. There is no visibility at national level on the amount of family planning commodities accessed by private sector providers through sub-county stores as these, along with vaccines, are issued by sub-county RH Coordinators. However, private sector respondents indicated that family planning commodities were increasingly unavailable and were limited to short-term methods suggesting that this reporting incentive was more diluted than it may have been in the past.

*“We used to get commodities from the government. It was based on our consumption data. Since COVID-19, we have not received any commodities.”* (Private sector respondent)

### Technical

***What is the contribution of the private sector to family planning? What are the assumptions underpinning adjustments in modelled estimates of contraceptive prevalence? Do these remain valid?***

 

*Summary*

Despite procedural challenges, private sector reporting rates have remained high improved over time however other data quality issues remain

Family planning services have not kept pace with the growth of private sector entities or their integration within government UHC schemes which may incentivise service provision through pharmacies

Secondary analysis for Nairobi County shows that the private facility contribution to family planning has declined over time while family planning access through pharmacies have grown over the same period; there is no visibility on this shift within the KHIS

Changes in private sector family service provision have implications for assumptions underpinning modern contraceptive modelled estimates and programmatic decision-making

**Figure 1.**
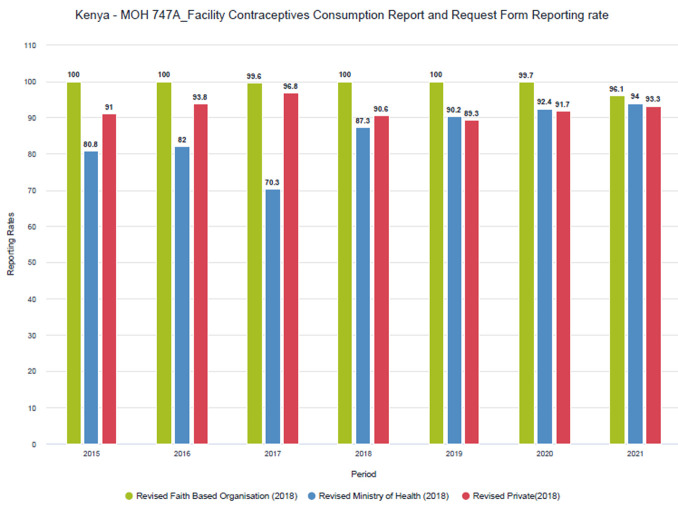
FCDRR reporting rates by sector (faith-based, public and private).

**Figure 2.**
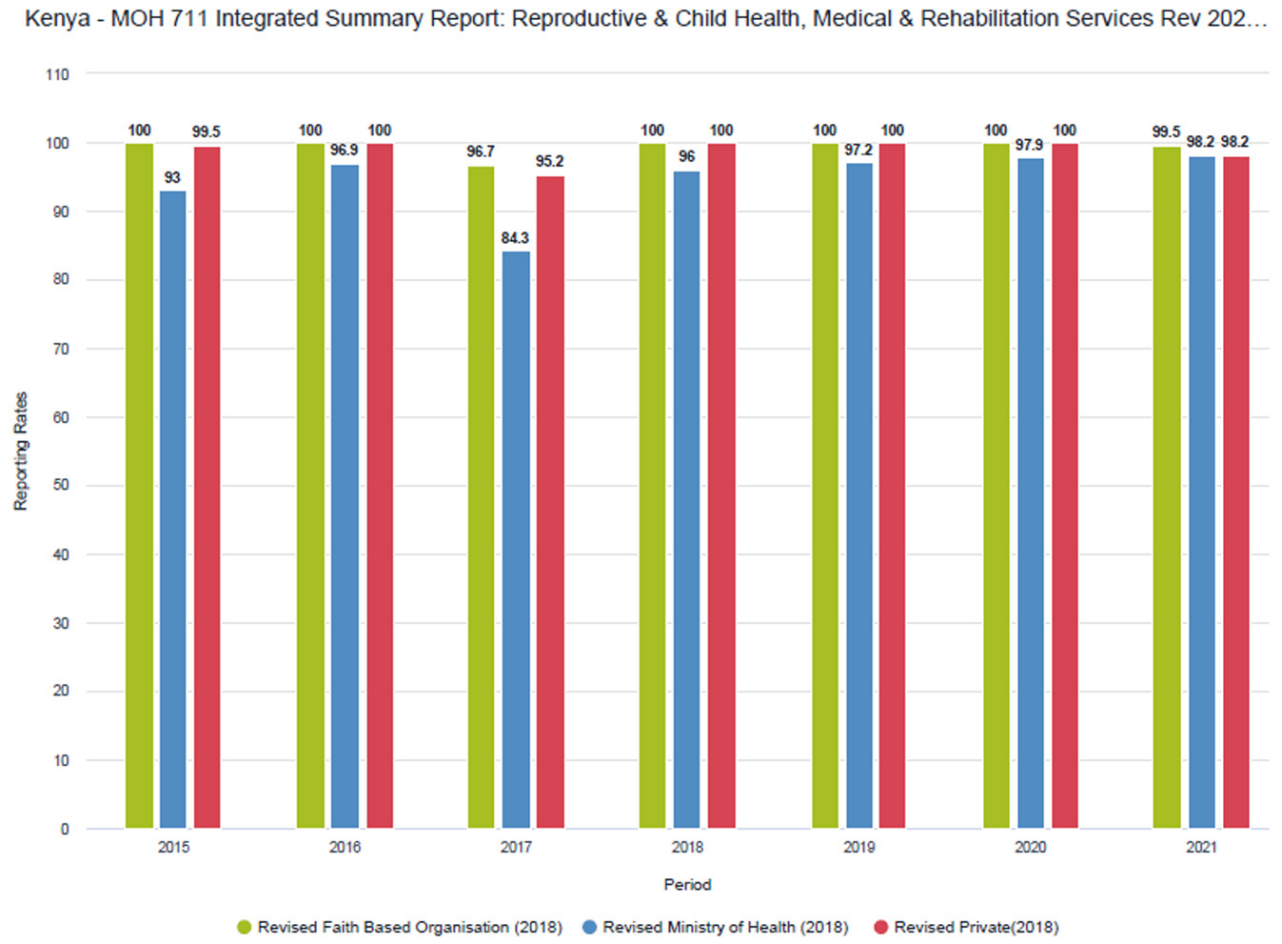
MoH 711 reporting rates by sector (faith-based, public and private).

Despite procedural challenges, private sector reporting rates have remained high over time ( Figure 1 and Figure 2). Improvements have been more pronounced in the MOH 711 as private facilities tend to complete the FCDRR only for the months when they received commodities from county depots. Within the MoH 711 there is more attention to data for maternal and child health (MCH), such as ANC and skilled delivery, as these services receive more scrutiny and attention.

While private sector reporting rates have remained high over time, other data quality issues remain. As some respondents indicated, incentives also exist for both over- and under-reporting family planning services. For example, commodity supply is based on the previous month’s consumption and may be inflated to gain more supply. Conversely, some providers may prefer to underreport so that family planning services remain modest and don’t attract attention (this may be in contexts where services are being provided to adolescents, given the current wave of conservatism and unclear communication from the MoH on adolescent access). For these and other reasons, data completeness and quality remain issues for family planning. While not specific to the private sector reporting, data quality has affected the ability to use family planning service statistics in the KHIS for modelled estimates of family planning. The private sector adjustment is not tied to reporting rates but assumes that if reporting is not 100%, then it is partial and equal to 50%. The question is whether this assumption is reasonable in general or is a different adjustment for urban counties needed where the private sector has a bigger impact on volumes.

**Figure 3.**
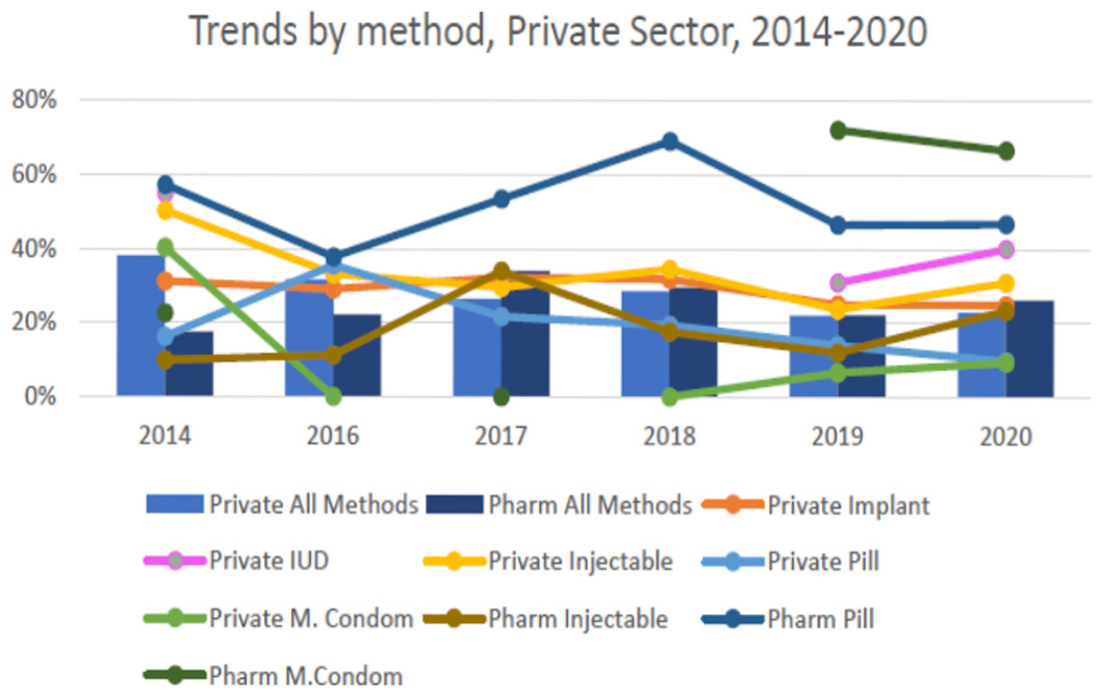
Trends in private sector family planning for Nairobi County.

Changes in private sector family planning services is supported by secondary analysis for Nairobi County ( Figure 3). This shows that the private facility contribution to family planning has declined over time. In contrast, services provided through pharmacies have grown over the same period. Pharmacy services are not captured in service statistics through the KHIS. There have been no concerted government-directed efforts in place to address the pharmacy governance gap for family planning ^4^.

Changes in private sector family service provision have implications for the assumptions underpinning mCPR modelled estimates. Gaps in understanding private sector contribution – both facility- and pharmacy-based – also have implications for programmatic decision-making. Accurate information is a powerful tool at both the county and national level when identifying and generating targets, allocating resources, and measuring performance. For instance, family planning forecasting and quantification in Kenya is based on consumption data yet it’s not clear how much the private sector is contributing to average monthly consumption. Furthermore, counties provide commodities to some private health facilities, but this is not accurately reflected in the national logistics information system.

Changes in private sector family planning services have not kept pace with the growth of the private health sector or their integration within government UHC schemes. While family planning services are included in Kenya’s essential health care package, this has not guided the development of NHIF packages or offer of family planning in practice through private health facilities ^5, 6^. In contrast, the contribution of pharmacies to family planning has grown – particularly during the COVID-19 pandemic – but with limited visibility in terms of contraceptive products, sources, quality, or volumes sold. A previous study noted the range of family planning products provided through pharmacies as including combined oral contraceptives, injectables, patches, spermicides and emergency contraception ^4^. This study attempted to support private pharmacies in Nairobi to report into the KHIS but struggled with pharmacy interest and sustained engagement.

## Conclusion

There is limited monitoring of the incentives and disincentives for reporting by private health facilities into the KHIS. These have changed over time and place and are summarised in Table 2. 

**Table 2.** Summary of incentives and disincentives for private sector reporting into the KHIS (KHIS = Kenya Health Information System; NHIF = National Hospital Insurance Fund).

Incentives	Disincentives
Recognition and respect for registry and reporting as a Government of Kenya policy requirement Accreditation within the NHIF and other insurance schemes Access to family planning commodities as part of submission of monthly reports	Lack of registers and reporting forms Manual entry of data and physical presentation of reports Lack of private entity visibility of facility data capture in the KHIS Maintenance of parallel systems, manual and digitised, within private facilities Family planning commodity stock outs

The study highlights the importance of routine monitoring and qualitative research methodologies as a complementary information source to data and information generated through the KHIS. Combined, these allow for richer understanding of “what we don’t know about private sector reporting”. Findings suggest that sustained private sector engagement is important to align incentives for reporting into the KHIS. Greater visibility on the role of pharmacies in family planning is further suggested. While pharmacies have been promoted as part of self-care, there is little visibility into the quality of products and services or the role that commodity stock outs in public and private health facilities play in increased client volumes through this channel. Respondents, both public and private, expressed concerns about the quality of services provided through pharmacies and chemists, in particular, in relation to the administration of injectables.

## Recommendations

Recommendations have been formulated for the Kenya MoH but may be applicable in other settings.

Clarify reporting of commodities on FCDRR with the intention of balancing this with client visits reported on the MoH 711. The FCDRR allows for reporting of all sources of commodities, not only those provided through KEMSA, and could provide useful information on private sector access to commodities, through KEMSA and the open market.

Consider the cost-benefit of paperless reporting into the KHIS by adopting digital versions of the MoH 711 and the FCDRR reporting forms. While these forms are seen as providing a paper trail for audit purposes, other means of verification could be adopted. In addition to being a ‘greener’ option, it is also more efficient. 

Gain greater insights into the provision of family planning through the pharmacies to understand the quality and volume of services and products offered through this channel. Additionally, explore the sources of private health facility commodities on the open market. This intelligence would help with forecasting and allocating scarce family planning resources as well as understand movement of commodities between the public and private sectors.

## Data Availability

This study uses secondary data from the Demographic and Health Surveys and Performance Monitoring for Action which provide anonymized data to researchers. The Demographic and Health Surveys are available from https://dhsprogram.com/ at no cost for academic research. Users must register to request and download dataset at https://dhsprogram.com/data/new-user-registration.cfm. After approval, datasets can be downloaded from https://dhsprogram.com/data/Using-Datasets-for-Analysis.cfm. Datasets are available as SAS, Stata, SPSS, and Flat Ascii files. The Performance Monitoring for Action surveys are available from https://www.pmadata.org/ at no cost for academic research. Users must register to request and download dataset at https://www.pmadata.org/data/request-access-datasets. Datasets are available as Stata files. Kenya Health Information System (log-in credentials required) https://hiskenya.org/ 1. Click the “data visualizer app” in the menu APP 2. Select dimensions i.e. Data, Period, Organization unit and Facility Ownership Data type: select “Data sets”, select “Reporting rates” Search and select “MOH 747A_Facility Contraceptives Consumption Report and Request Form Reporting rate Revised Private (2018) 2020” Period: Click “fixed period”, select “2015, 2016, 2017, 2018, 2019, 2020 and 2021” Organization Unit: Click “Organization Unit” and select “Kenya” Facility Ownership: Click “Facility Ownership” and select “Revised faith based organization (2018), revised Ministry of Health (2018), Revised private (2018)” 3. Click “Update” 4. Click “Options” then “Styles” to name title and axes 5. Click “Save” and Download” to save the chart in the computer. Generating Reporting Rates Family Planning Visits Access to Kenya Health Information System Aggregate (log-in credentials required) https://hiskenya.org/ 1. Click the “data visualizer app” in the menu APP 2. Select dimensions i.e. Data, Period, Organization unit and Facility Ownership Data type: select “Data sets”, select “Reporting rates” Search and select “MOH 711 Integrated Summary Report: Reproductive & Child Health, Medical & Rehabilitation Services Rev 2020 Reporting rate Revised Private(2018) “ Period: Click “fixed period”, select “2015, 2016, 2017, 2018, 2019, 2020 and 2021” Organization Unit: Click “Organization Unit” and select “Kenya” Facility Ownership: Click “Facility Ownership” and select “Revised faith based organization (2018), revised Ministry of Health (2018), Revised private (2018)” 3. Click “Update” 4. Click “Options” then “Styles” to name title and axes 5. Click “Save” and Download” to save the chart in the computer. For the qualitative research, detailed notes have not been made publicly available as there is the possibility that they would identify respondents. Please contact Gabrielle Appleford on gabrielle.aline@gmail.com for access. figshare: Private sector guides.docx. https://doi.org/10.6084/m9.figshare.21201193.v1 ^7^ This project contains the interview guide. Data are available under the terms of the Creative Commons Attribution 4.0 International license (CC-BY 4.0).

## Footnotes

^1^ Table 1 includes all facilities, not specifically those that provide family planning services.

## Software availability

Source code available from: https://github.com/Track20/NairobiPrivateSectorFPProvision.

Archived DOI at time of publication: https://doi.org/10.5281/zenodo.7086477 ^8^.

Zenodo: V1Track20/NairobiPrivateSectorFPProvision: Release 091622 (Track20, 2022).

This project contains the following files:

- Kenya Nairobi Method Source 091622.R
- LICENSE
- README.md

Code is available under an MIT license.
