# Supplementary material for: Incentives and disincentives to private sector reporting on family planning in Kenya: why these matter, and how they may change over time
Source: Gates Open Res. 2023 Aug 7;6:124. Originally published 2022 Oct 5. [Version 2] doi: 10.12688/gatesopenres.13909.2 (PMC10520241; doi:10.12688/gatesopenres.13909.2)
Supplement: Peer review history

# Reviewer report: Reviewer response for version 2

**DOI:** 10.21956/gatesopenres.16227.r34898

**Published:** 2023-09-25

**Authors:** Dominic Montagu

**Affiliations:** 1 Metrics for Management, Baltimore, MD, USA

The article provides a useful and new assessment of data reporting incentives and accuracy for family planning in Kenya. While some of the conclusions are difficult to be sure of given the limited variation in presented data (see Figure 2 and prior reviewer comments) and the inherent limits of the methodology; nevertheless the conclusions appear well founded and are of interest to policy makers. 

 The article has a number of limitations which future research will hopefully address.  Notably, while the private sector is defined in the methods section as including nursing homes, hospitals, and pharmacies, and the last is particularly important given the trending growth in pharmacy-delivered FP in Kenya, nevertheless interviews from pharmacies, hospitals, and nursing homes does not appear to have been collected. The number of private providers interviewed was small and restricted to providers affiliated with a large international NGO. This seems unduly narrow given the paper's desire to report on incentives for the private sector in aggregate. In light of the recommendation for governments to collect pharmacy data, what are the specific incentives or disincentives for private pharmacies to report to the government?  Might incentives for non-NGO-affiliated private providers, clinics, hospitals etc be different from the incentives reported by the private sector representatives interviewed?

 The literature on provider incentives for data reporting is not large, but the issue has been taken up before - for example by the WHO advisory group on governance of the private sector (2020 report); Kroll et al 2016: Involving private healthcare practitioners in an urban NCD sentinel surveillance system; Ahmadi et al, 2013: Disease Surveillance and Private Sector; Bhattacharyya et al 2016: District decision-making for health in low-income settings: a case study of the potential of public and private sector data in India and Ethiopia; Khoubati et al 2018; and a number of others others. 

 Background notes from the literature could have helped provide context for this study and would have underscored the how the authors' recommendations are likely to be applicable in settings beyond Kenya. 

 Despite these limitations, it is because the recommendations are likely to be applicable in settings beyond Kenya that the study is a valuable addition to the field.

Is the work clearly and accurately presented and does it cite the current literature?

Partly

If applicable, is the statistical analysis and its interpretation appropriate?

Not applicable

Are all the source data underlying the results available to ensure full reproducibility?

Yes

Is the study design appropriate and is the work technically sound?

Yes

Are the conclusions drawn adequately supported by the results?

Yes

Are sufficient details of methods and analysis provided to allow replication by others?

Yes

Reviewer Expertise:

Health systems governance, private healthcare provisions in Low and Middle Income Countries, quality of care in Low and Middle Income Countries.

I confirm that I have read this submission and believe that I have an appropriate level of expertise to confirm that it is of an acceptable scientific standard.

# Reviewer report: Reviewer response for version 1

**DOI:** 10.21956/gatesopenres.15202.r33281

**Published:** 2023-05-31

**Authors:** Ayub Manya

**Affiliations:** 1 Ministry of Health, Nairobi, Kenya

**Methods:**

 I suggest a rearrangement of the method section. Maybe it is not interesting to start with Ethics and consent. Regarding approval, some people might argue that the Ministry of Health does not have a scientific committee to approve research. Most of these are in KEMRI and teaching institutions like the University of Nairobi and Kenyatta University. However, the ministry can grant permission for the study once relevant institutions have done appropriate ethical reviews.

**Study Design:**

 Usually the design includes cross-sectional, case studies, cohort etc. But you have talked about data collection methods “The qualitative research entailed group discussions with county health teams in seven counties”. Please reorganize the study design section. In this area of the study design, the authors say that no literature review was done but contradicted themselves by saying “Background articles relevant to the context have been referenced in the paper”. In my opinion, this is part of the literature review.  

**Study site and population:**

 It is unclear how the counties and participants were selected. The only reason given was for Nairobi -“The four Nairobi sub-counties were selected based on contextual factors, such as the presence of large informal settlements and family planning organisations”.

**Results:**

 There is a tendency to provide discussion in the results section. Results should be in the results section without much explanation. Some parts of the results should have been in discussion to provide context. For instance: “Devolution, which commenced in 2013, transferred responsibility for service delivery to Kenya’s 47 counties". 

 I suggest that we separate the findings from the discussion.

**Conclusions:**

 The study is rich in results that are not consistent with the title. For instance, pharmacies were introduced but just lost focus. Given time, this would produce another paper with a different focus.

Is the work clearly and accurately presented and does it cite the current literature?

Yes

If applicable, is the statistical analysis and its interpretation appropriate?

Not applicable

Are all the source data underlying the results available to ensure full reproducibility?

Yes

Is the study design appropriate and is the work technically sound?

Yes

Are the conclusions drawn adequately supported by the results?

Yes

Are sufficient details of methods and analysis provided to allow replication by others?

Yes

Reviewer Expertise:

The authors to do some revisions as suggested

I confirm that I have read this submission and believe that I have an appropriate level of expertise to confirm that it is of an acceptable scientific standard.

# Reviewer report: Reviewer response for version 1

**DOI:** 10.21956/gatesopenres.15202.r33287

**Published:** 2023-05-30

**Authors:** Mai P. Do

**Affiliations:** 1 School Of Public Health And Tropical Medicine, Tulane University, New Orleans, Louisiana, USA

The paper addresses a very interesting topic related to the private sector’s involvement in FP service provision, and the quality of data reporting in this sector. I think the approach from three angles: governance, procedural and technical, is a good way to organize the study. However, I feel that the authors fell a bit short of delivering key messages, particularly to readers who may not have intimate understanding of the health systems and FP service provision in Kenya. Below are some suggestions for consideration:

1. Overall I think the “incentives” part is not well and explicitly laid out, relative to the “disincentives” part. It leaves a lot for readers to figure out on their own. This is particularly true for the section on governance, where I could not tell what may be incentives or disincentives. It is also a bit muddy in the other sections.
2. Related to the point above, it may be helpful to present the findings as public vs. private sector, and/or findings from public vs. private sector respondents. Only until much later in the paper that I understood that the private sector did not include pharmacies, when there was a discussion about changes in FP services in the private sector.
3. An overview of the FP service provision systems and environment in Kenya would help readers understand the findings better.
4. Similarly, the authors should define the public vs. private sector, counties, and explain KHIS as well as KHMFL in the Methods section.
5. It is not clear if Table 1 included only FP facilities or all facilities.
6. I am not sure if I agree that the private sector reporting rates have improved over time, based on Figure 1.
7. Under the technical section, I am not clear about the point of the discussion on the private sector adjustment. What adjustments and assumptions are being discussed? How do they influence the private sector facilities’ reporting and/or the quality of reporting?
8. The three paragraphs discussing changes in private sector that came right before Conclusions did not seem to come from the findings, but seemed more appropriate for a Discussion section.
9. Inconsistent use of “FP service provision” vs. “family service provision” – these are two different types of services.
10. Careful editing is necessary, e.g. “A literature review as not conducted as part of this study” as opposed to “…was not conducted…”.

Is the work clearly and accurately presented and does it cite the current literature?

Partly

If applicable, is the statistical analysis and its interpretation appropriate?

Not applicable

Are all the source data underlying the results available to ensure full reproducibility?

No source data required

Is the study design appropriate and is the work technically sound?

Yes

Are the conclusions drawn adequately supported by the results?

Partly

Are sufficient details of methods and analysis provided to allow replication by others?

Yes

Reviewer Expertise:

family planning

I confirm that I have read this submission and believe that I have an appropriate level of expertise to confirm that it is of an acceptable scientific standard, however I have significant reservations, as outlined above.
